# Artificial intelligence in atrial fibrillation: emerging applications, research directions and ethical considerations

**DOI:** 10.3389/fcvm.2025.1596574

**Published:** 2025-06-24

**Authors:** Ibrahim Antoun, Ahmed Abdelrazik, Mahmoud Eldesouky, Xin Li, Georgia R. Layton, Mustafa Zakkar, Riyaz Somani, G. André Ng

**Affiliations:** ^1^ Department of Cardiology, Glenfield Hospital, University Hospitals of Leicester NHS Trust, Leicester, United Kingdom; ^2^ Department of Cardiovascular Sciences, Clinical Science Wing, Glenfield Hospital, University of Leicester, Leicester, United Kingdom; ^3^ Department of Cardiac Surgery, Glenfield Hospital, University Hospitals of Leicester NHS Trust, Leicester, United Kingdom; ^4^ Department of Research, National Institute for Health Research Leicester Research Biomedical Centre, Leicester, United Kingdom; ^5^ Leicester British Heart Foundation Centre of Research Excellence, Glenfield Hospital, Leicester, United Kingdom

**Keywords:** atrial fibrillation, artificial intelligence, machine learning, ECG, risk stratification, remote monitoring, personalised medicine

## Abstract

Atrial fibrillation (AF) is the most prevalent sustained arrhythmia and a major contributor to stroke and heart failure. Despite progress in management, challenges persist in early detection, risk stratification, and personalised treatment. Artificial intelligence (AI), especially machine learning (ML) and deep learning (DL), has emerged as a transformative tool in AF care. This scoping review examines the applications of AI across key domains: detection, risk prediction, treatment optimisation, and remote monitoring. AI-driven models enhance AF detection by analysing ECGs and wearable device data with high accuracy, enabling early identification of asymptomatic cases. By incorporating diverse clinical, imaging, and genomic data, predictive models outperform conventional risk scores in estimating stroke risk and disease progression. In treatment, AI assists in personalised anticoagulation decisions, catheter ablation planning, and optimising antiarrhythmic drug selection. Furthermore, AI-powered remote monitoring integrates wearable-derived insights with real-time decision support, improving patient engagement and adherence. Despite these advances, significant challenges persist, including algorithm transparency, bias, data integration, and regulatory hurdles. Explainable AI (XAI) is crucial to ensure clinician trust and facilitate implementation into clinical workflows. Future research should focus on large-scale validation, multi-modal data integration, and real-world AI deployment in AF management. AI has the potential to revolutionise AF care, shifting from reactive treatment to proactive, personalised management. Addressing current limitations through interdisciplinary collaboration will be key to realising AI's full potential in clinical practice and improving patient outcomes.

## Introduction

Atrial fibrillation (AF) is the most common sustained arrhythmia worldwide, posing significant public health and clinical challenges due to its associated risks of stroke, heart failure, and mortality (1–3). AF management can be challenging, especially in the developing world, which has limited resources (4–11). However, AF is often intermittent and asymptomatic, making timely diagnosis challenging (12).

Artificial intelligence (AI) is the simulation of human intelligence in computers or machines, enabling them to perform tasks that typically require cognitive functions such as learning, reasoning, and problem-solving (13). Within AI, machine learning (ML) denotes a subset where algorithms learn from data to make predictions or decisions without being explicitly programmed (14). For example, ML can identify electrocardiogram (ECG) data patterns to distinguish between normal rhythm and atrial fibrillation. A more specialised subset of ML is deep learning (DL), which utilises layered neural networks inspired by the human brain. These models can process complex and high-dimensional data, such as continuous ECG signals or imaging data, and are particularly adept at uncovering subtle features that may be imperceptible to human clinicians.

AI techniques are increasingly employed in AF care to improve diagnostic accuracy, risk stratification, and treatment personalisation. For instance, DL algorithms have been shown to detect paroxysmal AF from sinus rhythm ECGs by learning subclinical signatures invisible to traditional analyses. Thus, a foundational understanding of these AI categories is essential for appreciating their applications in AF management, as explored throughout this review.

Over the last decade, AI has gained considerable momentum and is quickly becoming a mature discipline (15, 16). McCarthy coined the term AI in the late 1950s to denote the simulation of human intelligence in machines (17). Therefore, AI is not necessarily a newcomer, although most of its recent popularity is due to machine learning (ML). ML is a branch of AI that develops algorithms that use data to make predictions and improve their accuracy without being explicitly programmed to do so (18).

AI–particularly ML and DL techniques – have shown promise in improving AF detection, risk assessment, and management (19, 20) in recent years. Researchers are exploring AI across the spectrum of AF care, from early diagnosis using ECGs and wearables to personalised treatment selection. Despite encouraging results, there remain significant gaps in the literature and barriers to clinical implementation. Due to substantial advantages in big data processing, the use of AI in cardiovascular fields has recently aroused much attention. The use of AI in AF research has also increased significantly since 2012 (21).

The following article provides a structured overview of key research areas, highlighting current advances, unmet needs, and potential methodologies for future exploration.

## AI-driven early detection and diagnosis

**Figure 1:**
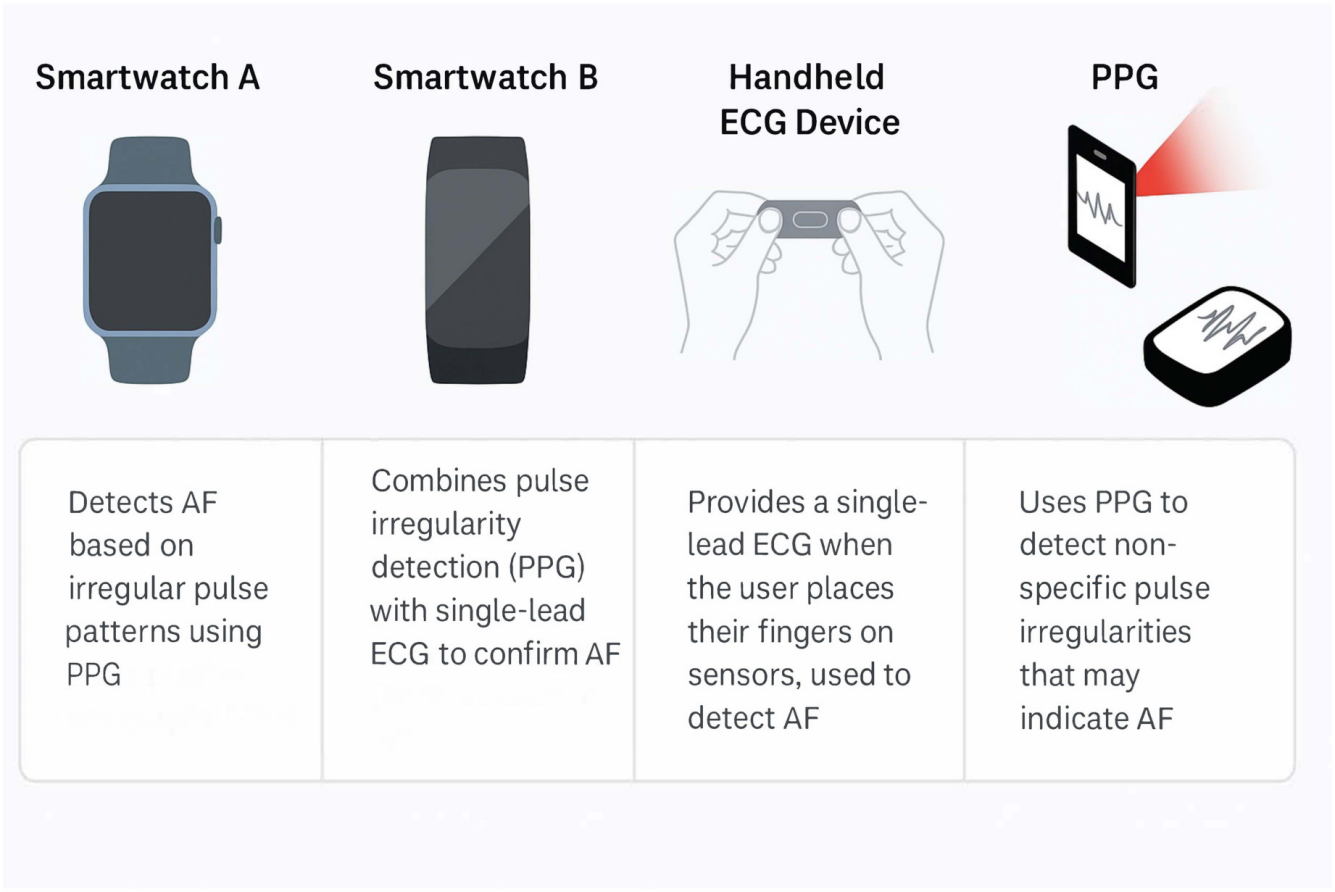
Some of the market's most used wearable devices to diagnose atrial fibrillation and their mechanism of detection. PPG, photoplethysmography; AF, atrial fibrillation; PPG; photoplethysmography.

AI algorithms can greatly enhance the early detection of AF by analysing large volumes of heart rhythm data from ECGs and wearable devices (22). Some currently available wearable devices are demonstrated in Figure 1. A traditional while the 12-lead ECG is considered the diagnostic gold standard for confirming AF, its sensitivity is limited in detecting paroxysmal AF (23). This is because the 12-lead ECG captures only a brief moment of cardiac electrical activity; thus, if the arrhythmia is not active during the recording, it may be missed. In contrast, wearable devices and continuous monitors can provide longer-duration rhythm surveillance, increasing the likelihood of detecting intermittent or asymptomatic episodes. ML models have shown high accuracy in detecting AF from single-lead ECGs or photoplethysmography (PPG) signals. For example, deep neural networks have achieved sensitivities and specificities in the 90%–99% range for classifying AF vs. normal rhythm using wearable ECG or PPG inputs (24, 25). Smartwatches equipped with FDA-cleared AF detection algorithms (using PPG and occasional ECG recordings) are increasingly popular and can reliably identify irregular pulse rhythms consistent with AF (26, 27). Notably, one smartphone-based PPG algorithm showed ∼89% sensitivity and ∼99% specificity compared to ECG diagnosis (28), highlighting the potential of ubiquitous devices for screening. This was also supported by a recent meta-analysis showing sensitivity of 92% and specificity of 96% in detecting AF on a single-lead ECG (29). AI can also detect subtle patterns in normal sinus ECGs that predict AF onset – in one study, an AI model predicted AF up to 4 h before an episode with an area under the curve (AUC) of 0.94. Another study of 180,922 patients showed that an AI-enabled ECG taken in normal sinus rhythm allows identification at the point of care of patients with AF (30).

Furthermore, the AI model predicts future episodes of AF with AUC 0.79 (0.72–0.86) in 122,394 patients, achieving the best prediction performance for males older than 70 years (31). These advances enable the detection of silent AF that would otherwise go unnoticed, allowing earlier interventions (e.g., starting anticoagulation to prevent stroke in asymptomatic patients) (32, 33). Despite promising accuracy, the real-world implementation of AI-driven AF detection faces challenges. Wearable-based algorithms can produce false positives or inconclusive alerts that require confirmatory traditional ECG (34–36). Many patients identified through smartwatch screening do not follow up with a physician, limiting clinical impact (37). Data from large digital health studies (Apple Heart Study and Huawei Heart Study) suggest that while technology can detect AF and even reduce stroke or hospitalisation rates when accompanied by proper follow-up, ensuring adherence to alerts by patients and providers is challenging (37, 38). There is also variability in performance across different devices and patient populations due to data quality and algorithm differences.

Future research may improve signal processing and ML robustness to reduce false alarms (for example, using ensemble models that combine ECG, PPG, and accelerometer data for contextual intelligence). Unsupervised learning might uncover new AF-related patterns (e.g., in heart rate variability) that aid earlier detection (39). Moreover, integrating wearable data into clinical workflows via secure health information systems can ensure that clinicians are notified of significant events (40). Developing patient engagement strategies (adaptive app notifications or health coaching chatbots) to encourage prompt action after an AF alert will also be crucial to translating early detection into improved outcomes. While early detection of AF through wearable or implantable devices offers clear benefits, such as stroke prevention and timely intervention, it also raises concerns about overdiagnosis and overtreatment. The number needed to screen (NNS) to prevent one stroke varies depending on the population risk profile and screening method, with estimates ranging from 83 to 300 in moderate-risk populations. However, this remains an area of ongoing investigation (41).

Moreover, treating every detected AF episode with anticoagulation, especially short, asymptomatic episodes, may not be warranted. Recent evidence from the NOAH-AFNET 6 and ARTESiA trials suggests that oral anticoagulation in patients with device-detected subclinical AF may not significantly reduce stroke risk and might increase bleeding risk, particularly in patients without other high-risk features (42, 43). These findings highlight that not all AF episodes carry equal clinical risk, and treatment decisions should consider AF burden, episode duration, comorbidities, and patient-specific stroke risk.

As such, integrating AI-powered monitoring must complement clinician oversight and risk-based interpretation rather than automatically escalate to treatment. Future AI tools may help stratify the most clinically relevant episodes, aligning screening with personalised therapeutic thresholds. Many AI models using wavelets and ECG signals have shown high classification performance in AF detection, often exceeding 95% accuracy. These models vary in complexity and validation methods, as summarised in Table 1. High-quality studies, including large prospective cohorts and randomised trials assessing wearable-based AF detection, are summarised in Table 2.

**Table 1:** Performance of selected major AF classification studies using wavelets and AI on ECG.

Study and year	Year	AI model	Evaluation method	Accuracy
Xu et al., (44)	2021	SVM-KNN-DT-ELM	Fold cross validation	98.6%
Singh et al., (45)	2020	SVM-LSTM	Fold cross validation	99.4%
Abdullah et al. (46)	2020	CNN-LSTM	Fold cross validation	98.1%
Ullah et al. (47)	2020	2-D CNN	Fold cross validation	99.1%
Zhao et al. (48)	2020	DCNN	Fold cross validation	87.8%
Nurmaini et al. (49)	2020	CNN-RNN-DNNs	Fold cross validation	99.1%
Kora et al. (50)	2019	SVM-KNN	Not published	94%-99.5%
Chashmi et al. (51)	2019	NN-SVM	10-fold cross validation	99%
Alarsan et al. (52)	2019	DT-RF-GDB	Fold cross validation	98%
Anwar et al. (53)	2018	NN	3-fold cross validation	99.8%
Lassoued et al. (54)	2017	ANN-MLP and neurofuzzy	GD + Momentum	99%
Xin et al. (55)	2017	SVM	Not published	92%
Filos et al. (56)	2017	SVM	Not published	93.8%
Kaya et al. (57)	2017	DT-SVM-NN	Fold cross validation	98.9%-99.3%
Saraswat et al. (58)	2017	PNN	Fold cross validation	100%
Dewangan et al. (59)	2016	ANN	Not published	87%
Thomas et al. (60)	2015	ANN	Fold cross validation	94.6%
Barmase et al. (61)	2013	Markov	Fold cross validation	99.8%
Sarkaleh et al. (62)	2012	MLP-NN	Fold cross validation	96.5%
Kim et al. (63)	2011	ELM	Not published	97.9%

SVM, support vector machines; KNN, K-nearest neighbour; DT, decision trees; ELM, extreme learning machine; LSTM, long short-term memory; CNN, convolutional neural network; RNN, recurrent neural network; DNN, deep neural network; NN, neural network; RF, random forests; GBD, gradient-boosted trees; ANN, artificial neural network; MLP, multilayer perceptron; PNN, probabilistic neural network.

**Table 2:** Key evidence from high-quality studies on wearables for AF detection.

Study name and year	Study type	Device/technology	Sample size	Main findings
Apple Heart Study, (64)	Prospective cohort study	Apple Watch (PPG-based)	419,297	PPV: 84% for irregular rhythm notification indicating AF
Huawei Heart Study, (38)	Prospective cohort study	Huawei Watch (PPG-based)	246,541	PPV of irregular pulse notifications: 91.6%; confirmed AF in 87%
mSToPS Trial, (65)	RCT	Zio XT patch (ECG-based)	2,659	AF newly diagnosed in 6.3% of actively monitored vs. 2.3% in controls
REHEARSE-AF Trial, (66)	RCT	AliveCor Kardia (single-lead ECG)	1,001	AF detection significantly increased (3.8% vs. 1%) using wearable vs. routine care
SCREEN-AF Trial, (67)	RCT	AliveCor KardiaMobile ECG	856 aged ≥ years old	Increased AF detection in high-risk elderly patients (5.3% vs. 0.5% in routine care)
Fitbit Heart Study, (68)	Prospective cohort study	Fitbit PPG	455,699	High accuracy (PPV: 98%) for AF detection in individuals with irregular heart rhythm alerts
Huawei heart study, (69)	Prospective cohort study	Huawei PPG	246,541	PPV: 91.6%, Both suspected AF and identified AF markedly increased with age

AF, atrial fibrillation; ECG, electrocardiogram; PPG, photoplethysmography; PPV, positive predictive value; RCT, randomised controlled trial.

## AI for risk stratification and prediction of stroke or complications

Risk stratification is vital in AF to identify high-risk stroke, heart failure, or disease progression patients. Traditional clinical risk scores like CHA₂DS₂-VA are widely used for estimating stroke risk, but they have only modest discriminatory ability (C-statistic around 0.60) and ignore potentially important factors (70, 71). AI-based models offer a more individualised approach. Machine learning algorithms can ingest a wide range of clinical features – including demographics, comorbidities, lab results, imaging findings, and even social determinants of health – to predict adverse outcomes. Studies have shown that ML models outperform conventional scoring. For example, a convolutional neural network model achieved an AUC of 0.70 for predicting near-term stroke in AF patients, significantly better than the <0.50 AUC of CHA₂DS₂-VA in the same cohort. Researchers note that current scores are “rudimentary”, AI can capture additional risk predictors (like chronic kidney disease, prior heart failure, or socio-environmental factors) that improve accuracy (72, 73). Likewise, ML has been applied to predict other complications: one report used an AI algorithm on clinical and imaging data to identify patients likely to progress from paroxysmal to persistent AF or to develop heart failure (19). Another model could predict postoperative AF (a common surgical complication) with ∼87% sensitivity and 83% specificity, enabling prophylactic strategies (74).

## Stroke prediction and other outcomes

A major focus is AI use for stroke risk prediction in AF beyond standard scoring. ML models have been trained on extensive electronic health record datasets to calculate stroke risk automatically; these models can incorporate nuanced patterns (e.g., burden of AF, patterns of blood pressure control, or brain imaging markers) that static clinical scores cannot (75). Early results are encouraging, but validation is still required. Similarly, AI models have demonstrated the ability to predict AF recurrence after catheter ablation, guiding follow-up intensity and additional therapy. For instance, a DL algorithm using procedural data and patient characteristics predicted post-ablation AF recurrence with approximately 85% accuracy (76). Including advanced phenotypic data like left atrial fibrosis on magnetic resonance imaging (MRI) or anatomical features on cardiac computed tomography (CT) is a promising avenue to further refine such predictions (77).

While many AI risk models have been developed, prospective validation and clinical uptake are key gaps. Few have been tested in randomised trials to show improved patient outcomes or cost-effectiveness (19). Future research should emphasise external validation of AI risk tools across diverse populations to ensure generalizability. Creating large, standardised, and labeled datasets (potentially through multi-center or federated learning collaborations) will help address current data heterogeneity issues (78). Moreover, researchers are exploring explainable AI techniques to identify which risk factors drive predictions, which could reveal novel modifiable risk factors (for example, an explainable model highlighted how combinations of social and clinical factors influence stroke risk in AF (79, 80). Such insights might inform more holistic risk management strategies. In summary, AI-driven risk stratification promises to move beyond one-size-fits-all metrics toward nuanced, precision risk profiles for each AF patient.

## AI-assisted treatment optimization (medications and ablation planning)

Managing AF involves choices between rate vs. rhythm control, various antiarrhythmic drugs, anticoagulation strategies, and interventional therapies like catheter ablation. AI can assist clinicians in tailoring these decisions to individual patients. One emerging application uses ML to determine which patients benefit most from a given therapy. For example, an AI-based causal forest model was recently developed using data from over 700,000 AF patients to personalise stroke prevention therapy (81). This model analyses patient characteristics to decide whether a patient would have better outcomes with lifelong anticoagulation (using a direct oral anticoagulant, DOAC) or a left atrial appendage occlusion (LAAO) procedure. Notably, it can identify subgroups of patients for whom LAAO provides a positive net benefit (reducing stroke risk without undue bleeding risk) vs. those who fare better on medication (81). Such AI-driven decision support could fill an important gap in current practice, where selecting candidates for LAAO vs. DOAC is often unclear. Similarly, ML has been applied to recommend optimal rate or rhythm control strategy by predicting outcomes like symptom improvement or hospitalisation risk under each approach (82). However, more research is needed in this area. A significant gap is the lack of clinical trial evidence demonstrating that AI-guided treatment decisions improve long-term outcomes in AF. Ongoing studies will reveal the real-world impact, particularly in applying AI recommendations for therapy selection in a prospective trial. Methodologically, incorporating reinforcement learning could prove beneficial—an AI that “learns” the optimal treatment through trial and error on patient data might suggest dynamic treatment adjustments, such as escalating from drugs to ablation if specific patterns arise. Furthermore, integrating AI into electronic health records as a clinical decision support tool at the point of care is another avenue to explore, but it must be implemented in a way that aligns with the clinician's workflow and provides transparent reasoning for recommendations (83). Combining an AI's predictive power with a physician's clinical judgment may yield the best outcomes in tailoring AF therapy plans.

## AI role in catheter ablation

Catheter ablation is an effective rhythm-control therapy; success rates can differ, and repeat procedures are common (84). AI has the potential to enhance patient selection and procedural planning for ablation. An innovative study employed DL on cardiac imaging data to predict non-pulmonary vein (PV) triggers of AF before an ablation procedure (85). Typically, ablation targets triggers in the PVs, but patients with additional atypical trigger sites often experience recurrence. The AI model correctly predicted these non-PV trigger locations in ∼82% of cases (64% sensitivity, 88% specificity), improving the overall accuracy of identifying all trigger sites to 89% (85). This information can help electrophysiologists personalise the ablation strategy rather than using a uniform approach for all patients. AI has also been utilised in intra-procedural mapping – for instance, algorithms that rapidly interpret electrogram patterns to distinguish AF drivers or to titrate energy delivery. Early clinical experience with AI-guided ablation dosing, including high-power short-duration ablation with algorithmic monitoring for safety, shows the potential to reduce complications (86). Additionally, AI can assist in medication optimisation by predicting an individual's response or side-effect risk to a particular antiarrhythmic drug based on their profile, though this is still largely theoretical (87). Recent high-quality evidence from the TAILORED-AF trial supports using AI-guided ablation in persistent AF. This randomised, double-blind trial showed that targeting AI-identified spatio-temporal electrogram dispersion areas in addition to standard PVI significantly improved 12-month AF freedom rates (88% vs. 70%, *P* < 0.0001). While safety was comparable, procedure duration was longer. These results validate AI's role in refining ablation strategy but highlight the need for further trials to assess long-term outcomes, reproducibility, and workflow integration (88). New AI-based software solutions were designed to assist operators in targeting AF drivers. Acute and long-term outcomes suggest that the AI-based AF electrogram software delivers simple perioperative cues, ensuring standardisation across multiple platforms, catheters, and operators (89). For example, a recent study demonstrated that DISPERS-guided ablation using ML software (the Volta VX1 software) and PVI for long-standing persistent AF caused a lower risk of AF recurrence in long-term follow-ups (90).

## Machine learning models for personalized AF management

Personalised medicine in AF aims to move beyond generalised treatment guidelines and towards individualised care plans. AI is a key enabler of this vision, as it can analyse each patient's unique combination of factors. Holistic ML models can assimilate diverse data, including genomics, biomarkers, lifestyle factors, and detailed disease history, to define patient subgroups or “phenotypes” of AF. For instance, clustering algorithms (unsupervised ML) have been used to identify novel AF phenotypes that might respond differently to treatments (39). For example, a patient might have AF driven largely by obesity and hypertension (risk-factor-mediated AF) (91). At the same time, another's AF might be linked to specific genetic variants or fibrotic scar burden in the atrium. AI can help classify such subgroups, which is the first step to personalised therapy, including aggressive risk factor modification for one phenotype vs. early ablation for another.

Early rhythm control has been proven beneficial as AF begets AF, and early intervention has shown better results irrespective of the mechanisms. As there is an evolution of AF in many cases (triggers at the beginning with short episodes and fibrosis with persistent AF types), AI might help identify patients at risk for AF and help establish a primary preventive therapy. In case of AF, early treatment should be offered irrespective of risk factors, as e.g., those with heart failure benefit most. Researchers have proposed new AF classifications using ML, which are being studied for their prognostic and therapeutic relevance (92). An example of such translational work is the ARISTOTELES project, which uses AI to integrate clinical, imaging, genetic, and biomarker data to personalise risk prediction and treatment in patients with AF and multimorbidity. The project aims to refine stroke and bleeding risk stratification in the context of oral anticoagulation and to address therapeutic decision-making complexities in patients with coexisting conditions such as heart failure, diabetes, and chronic kidney disease. By embracing a holistic, data-driven approach, ARISTOTELES supports guideline-aligned, individualised care and seeks to improve outcomes, reduce adverse events, and optimise resource use in real-world clinical settings (93). Key studies exploring AI-based classification and prediction of AF, particularly those using ECG or clinical data for risk stratification, are outlined in Table 3.

**Table 3:** Main evidence on AI-assisted treatment in atrial fibrillation.

Study/Author (year)	AI Application	Population	Main findings
Ngufor et al. (81)	Personalized anticoagulation vs. LAAO	744,190 AF patients	AI model identifies optimal stroke prevention therapy, highlighting patients benefiting most from DOAC or LAAO
Kim et al. (94)	AI use in guiding rhythm management	≈ 42,000 AF patients	Healthcare systems using algorithms for AF rhythm management must balance prediction accuracy with model interpretability
Liu et al. (85)	Catheter ablation strategy	521 patients undergoing PAF ablation	DL predicted non-PV triggers with 82% accuracy (88% specificity, 64% sensitivity)
Deisenhofer et al. (TAILORED-AF Trial) (95)	AI-guided catheter ablation	370 persistent AF patients (AI tailored arm, *n* = 187)	AI-guided ablation plus standard PVI improved 12-month AF-free rates (88% vs. 70%, *p* < 0.0001)
Bahlke et al. (90)	ML-guided DISPERS ablation software	50 persistent AF undergoing ablation	ML-assisted ablation software reduced long-term recurrence rates compared to standard PVI
Sanchez de la Nava et al. (87)	Antiarrhythmic drug selection	127 AF patient models	AI predicted patient-specific drug responses in silico
Seitz et al. (89)	Standardization of ablation outcomes	85 persistent AF patients	AI software standardized electrogram-based ablation across multiple operators and platforms

AF, atrial fibrillation; AI, artificial intelligence; DL, deep learning; DOAC, direct oral anticoagulants; LAAO, left atrial appendage occlusion; ML, machine learning; PVI, pulmonary vein isolation; PV, pulmonary vein; PAF, paroxysmal atrial fibrillation.

## Integration of AI with genomic and clinical data

In the era of precision medicine, merging AI with genomics presents a promising methodology. Extensive genome-wide association studies have identified numerous genetic loci associated with AF, yet interpreting these for individual risk remains complex. AI can bridge this gap by integrating genetic risk scores with phenotypic data. For instance, one analysis demonstrated that AI-driven ECG analysis serves as a practical and cost-effective means of predicting AF risk and onset, capturing lifetime cardiac variations, whereas genomics offers a more static risk profile; the combination of the two facilitates “truly individualised care” that transcends the average patient model (96).

This might mean an AI model uses a patient's ECG and blood biomarkers to detect subtle signs of atrial remodelling while incorporating their genetic predisposition to refine risk and guide preemptive therapy (97). However, a meta-analysis indicated that AF prediction using AI is still underdeveloped, though DL techniques are becoming increasingly accurate. Nevertheless, these methods are not being applied as frequently as expected (98).

## Challenges and research opportunities

Achieving personalised AF management with AI faces several hurdles. One is data silos – the need to gather comprehensive datasets that include outcomes of different management strategies in diverse patient profiles. Collaborative consortia and data-sharing with privacy protections could help amass enough data for robust personalised models. Another challenge is interpretability: Clinicians will require understandable explanations for why an AI recommends a personalised approach. Research into explainable AI for personalised medicine is, therefore, critical (99, 100). Furthermore, prospective trials are needed to test AI-guided personalised management: for example, an algorithm might propose varying treatment strategies for patients based on their cluster phenotype – testing this against usual care will show if personalisation via AI improves outcomes like AF recurrence or quality of life. If successful, these approaches could pave the way to truly precision cardiology, where every AF patient's management is dynamically tailored by AI insights drawn from patients “like them” in large databases.

## The role of AI in remote monitoring and patient adherence

Remote monitoring technologies for AF allow continuous or frequent rhythm surveillance outside the clinic. AI plays a vital role in interpreting the large volume of data generated by remote monitoring devices. Smartwatches and patches can detect arrhythmias in real time, but AI algorithms must distinguish true AF episodes from noise or benign irregularities (101). When deployed effectively, AI-driven remote monitoring can alert clinicians to AF onset or recurrence, enabling earlier intervention (68). Studies have demonstrated that mobile health interventions for AF can reduce healthcare utilisation combined with algorithmic monitoring. In one cluster trial, patients supported by a mobile app and wearables had significantly lower rehospitalisation and adverse event rates than those with standard care (38). Another study showed that contactless AI monitoring could accurately detect AF without any wired device (102, 103). This sets the stage for futuristic remote surveillance methods that are seamless for patients.

A critical aspect of remote AF management is ensuring patients adhere to monitoring and therapy. AI can assist here through personalised feedback and coaching. For example, smartphone apps with a conversational “relational agent” have been piloted to engage AF patients daily, provide education, and encourage medication adherence. In a 120-patient trial, those randomised to a 30-day smartphone app with an AI-driven virtual coach and a portable ECG monitor showed significant improvements in adherence and quality of life compared to controls (104). This suggests that AI can help close the gap between detecting AF and prompting patients to act, including taking medications and contacting healthcare providers. Nonetheless, challenges remain: Large-scale screening studies found that some people ignored or delayed responding to AF alerts on their devices (37). This highlights that technology alone is insufficient; behavioral science must be integrated into AI systems.

Future research should explore adaptive notification systems that adjust the urgency and style of alerts based on patient behavior patterns to avoid alarm fatigue while conveying importance (105). AI might predict which patients are at risk of non-adherence. This can be done by analysing their past application usage, heart rate trends, or speech patterns in consultations (106). Combining remote monitoring AI with telemedicine services is another promising avenue. If an algorithm detects AF, it could automatically schedule a telehealth visit or message a healthcare provider, streamlining the response. AI can turn passive remote monitoring into an active, responsive system that detects AF, facilitates prompt management, and keeps patients engaged in their care.

## Explainability and ethical concerns in AI-based AF detection and treatment

Many AI models, especially DL, are often criticised as “black boxes” – they make predictions (AF detected or stroke risk high) without an easily interpretable rationale. In the context of AF, lack of explainability can hinder clinician trust and adoption. Researchers have started integrating explainable AI (XAI) techniques into their models to address this. For instance, one study converted PPG pulse data into images and used a convolutional neural network to classify AF; importantly, they incorporated XAI methods to highlight which signal features contributed to the classification, providing transparency to clinicians (107). The resulting model achieved 100% accuracy in distinguishing AF from normal rhythm while ensuring the decision process was interpretable. Such approaches allow physicians to verify that the AI detects physiologically relevant patterns (like irregular RR intervals or fibrillatory waves) rather than spurious noise. Explainability is equally crucial in AI-driven treatment recommendations – doctors need to understand why an algorithm favors a particular therapy for a patient (perhaps due to that patient's combination of age, stroke risk, and prior haemorrhage history) to feel comfortable following the advice (108). Developing user-friendly visualisation tools and explanation summaries for AI outputs is an active area of research that will make AI more ethically and clinically palatable.

The use of AI in AF raises several ethical considerations. Patient data privacy is paramount, as AI models often require large datasets (ECGs, wearable records, and health records) that may contain sensitive information. Ensuring compliance with privacy regulations and using data anonymisation or federated learning (where data stay at hospital sites and only model updates are shared) can mitigate privacy risks (73). Bias is another concern – if an AI model is trained mostly on certain demographics, it may perform less accurately for underrepresented groups, potentially exacerbating healthcare disparities. For example, an algorithm trained predominantly on younger patients might miss AF in the elderly or vice versa. Researchers have pointed out that algorithms must consider social determinants of health and diverse patient attributes to avoid bias (109). Ongoing efforts to use diverse training datasets and to audit algorithms for fairness are critical (110). Additionally, there is the ethical question of handling false positives/negatives: a false positive AF alert can cause anxiety and unnecessary testing, while a false negative might give false reassurance. Striking the right balance in algorithm sensitivity is partly a clinical value judgment. Some ethicists have raised concerns about widespread consumer AF screening being promoted without clear guidance, potentially putting users at risk of over-treatment or anxiety for the sake of tech company marketing (111, 112).

Several strategies are recommended to ensure that AI in AF is used responsibly. First, ethicists and patient representatives should be involved early in developing AI tools to identify concerns such as consent and data ownership. Second, incorporate the core principles of biomedical ethics: beneficence (the AI should demonstrably assist patients), nonmaleficence (minimising harm from errors), autonomy (patients should control how their data are used and be informed about AI's involvement in their care), and justice (equitable access to the benefits of AI) (113, 114). Specifically, this could mean providing patients with a straightforward opt-in/out option for data sharing and ensuring that AI tools are accessible in community hospitals, not just academic centres. Third, maintain a human-in-the-loop approach: AI should support, rather than replace, clinician decision-making, and clinicians should override or question AI when it conflicts with clinical judgment or patient preferences (115). The medical community can harness their advantages while upholding high ethical standards by making AI systems transparent, secure, and patient-centred. AI-driven precision medicine in AF must move beyond purely clinical or algorithmic outputs to incorporate patients' own perceptions of safety and autonomy—factors deeply influenced by cultural background, previous healthcare experiences, and psychosocial context. Overreliance on binary risk models or population-level predictions risks marginalising patient values and eroding trust. Ethically grounded AI must therefore embed principles of shared decision-making, allowing patients to weigh algorithmic recommendations against their personal goals and beliefs. This includes providing transparent explanations of AI outputs and fostering cultural competence in both data design and clinical implementation to support genuinely patient-centred care.

## Challenges in implementing AI for AF in clinical practice

Despite the increasing research, there is a recognised gap between AI models developed in laboratories and the tools that physicians use at the bedside. One significant challenge is rigorous clinical validation (116). Many AI algorithms for AF detection or risk prediction have been tested retrospectively or on limited datasets (117, 118); few have been evaluated in prospective clinical trials or real-world practice settings. Without evidence that AI improves patient outcomes or workflow, healthcare providers may hesitate to adopt these tools. Regulatory approval pathways for AI in medicine are evolving – algorithms may require clearance as medical devices, and there are questions about how to regulate AI systems that continuously learn and update. Obtaining regulatory approval can be complex and time-consuming, particularly if an AI's decision-making logic is not easily interpretable to regulators.

Implementing AI in daily practice also presents logistical challenges. Hospitals must integrate AI software with electronic health record systems and device data streams, ensuring reliability and cybersecurity. Clinicians experience alert fatigue from existing monitoring systems; introducing AI alerts or recommendations could further burden them if not carefully designed (119). Therefore, human factors engineering is essential – AI tools must be intuitive, with concise and relevant outputs. Clinician training is another crucial aspect: cardiologists and general practitioners will require a fundamental understanding of how the AI operates and its limitations to use it effectively and maintain trust in the system (120). At the practice level, some resistance to new technology is natural; early adopters must advocate for successful use cases to persuade their peers of AI's potential value. High-quality data is the fuel for AI, and data can often be messy in practice. AF-related data frequently resides in disparate sources and may lack standardisation. As noted in the review article by Popat et al., variability and heterogeneity in data and methods have led to the inconsistent performance of AI tools across studies (73). This suggests that an AI model may not generalise effectively without standard data formats and solid data governance when implemented in a different hospital or demographic. Initiatives such as establishing shared data repositories and adopting common standards for documenting arrhythmia data can be beneficial. Another obstacle is the computational infrastructure – not all clinics can execute advanced AI algorithms in real time. Cloud-based solutions could alleviate this issue; however, data security and latency concerns may occur.

To bridge these gaps, researchers and healthcare systems are beginning to collaborate on implementing science for AI. This includes pilot programmes where AI tools are introduced in a controlled manner, and their impact on decision-making, outcomes, and clinician workload is measured. Feedback from these pilots can guide iterative improvements. Additionally, clear guidelines from professional societies on how to incorporate AI into AF management (when to trust an AI-detected AF episode or how to use an AI risk score in anticoagulation decisions) will provide reassurance and standardisation (121). Addressing implementation challenges will ultimately require a multidisciplinary approach, with data scientists, information technology specialists, clinicians, and administrators working together to ensure that AI for AF is accurate, useful, and seamlessly embedded in care delivery.

## Future directions for AI research in AF

The intersection of AI and AF management is a rapidly evolving field with several exciting avenues for future research to address current gaps. A clear need exists for developing standardised, well-annotated datasets for AF. Future research could focus on building large, shared databases of ECGs (including those in sinus rhythm and AF), patient outcomes, and imaging data, which would enable more robust model training and validation. International collaborations and data-sharing agreements, employing privacy-preserving techniques, will expedite this progress and minimise duplication of effort. Integrating various data modalities presents a promising research frontier. AF is a multi-factorial disease; thus, combining multi-modal data in AI models may yield new insights. For instance, researchers could develop models that input ECG signals, cardiac MRI scans, genetic information, lab results, and wearable activity logs to provide a comprehensive risk assessment or guide therapy. Initial efforts to combine ECG-based AI with genomics have demonstrated the potential for more precise predictions (96). Future studies will likely build on this by including proteomics or metabolomics to capture substrate changes in AF. Such comprehensive models could, for example, predict which patients will respond to upstream therapies, such as aggressive risk factor management or anti-inflammatory treatment, based on their unique biomarker signature.

With the proliferation of wearable devices, one future direction is to deploy AI algorithms that operate in real time on device data. A smartwatch could detect AF and predict when an episode is likely to occur based on recent trends, alerting the patient to take preventive actions, such as performing a breathing exercise or taking a pill (122). Research into lightweight AI models that can run on wearable hardware or smartphones for continuous monitoring without relying on cloud servers will be crucial for responsive and scalable AF management. Future AI research may also contribute to the development of new treatments for AF. By analysing large datasets of drug responses, AI could aid drug discovery by identifying novel molecules or repurposing existing medications that might effectively treat AF. An AI-driven “autopilot” for PVI is on the horizon. These include algorithms capable of guiding catheters during ablation with minimal human input, optimising lesion placement and duration in real time based on patient-specific atrial anatomy and electrophysiology (89). Preliminary steps in this direction, such as AI mapping of electrical rotors or lesion assessment through imaging, warrant further investigation exploration. As AI systems become more complex, a parallel research priority is enhancing their explainability and user trust. Future AI models for AF might incorporate built-in explanation modules that translate their complex computations into cardiologist-friendly explanations, highlighting which part of a P wave or what clinical factor led to a high-risk prediction. Measuring and improving clinician trust in AI recommendations will likely become a research topic, possibly by testing different explanation strategies in user studies. This also ties into medicolegal considerations – defining how responsibility is shared between AI tools and human providers – which may be informed by research in ethics and legal frameworks.

**Figure 2:**
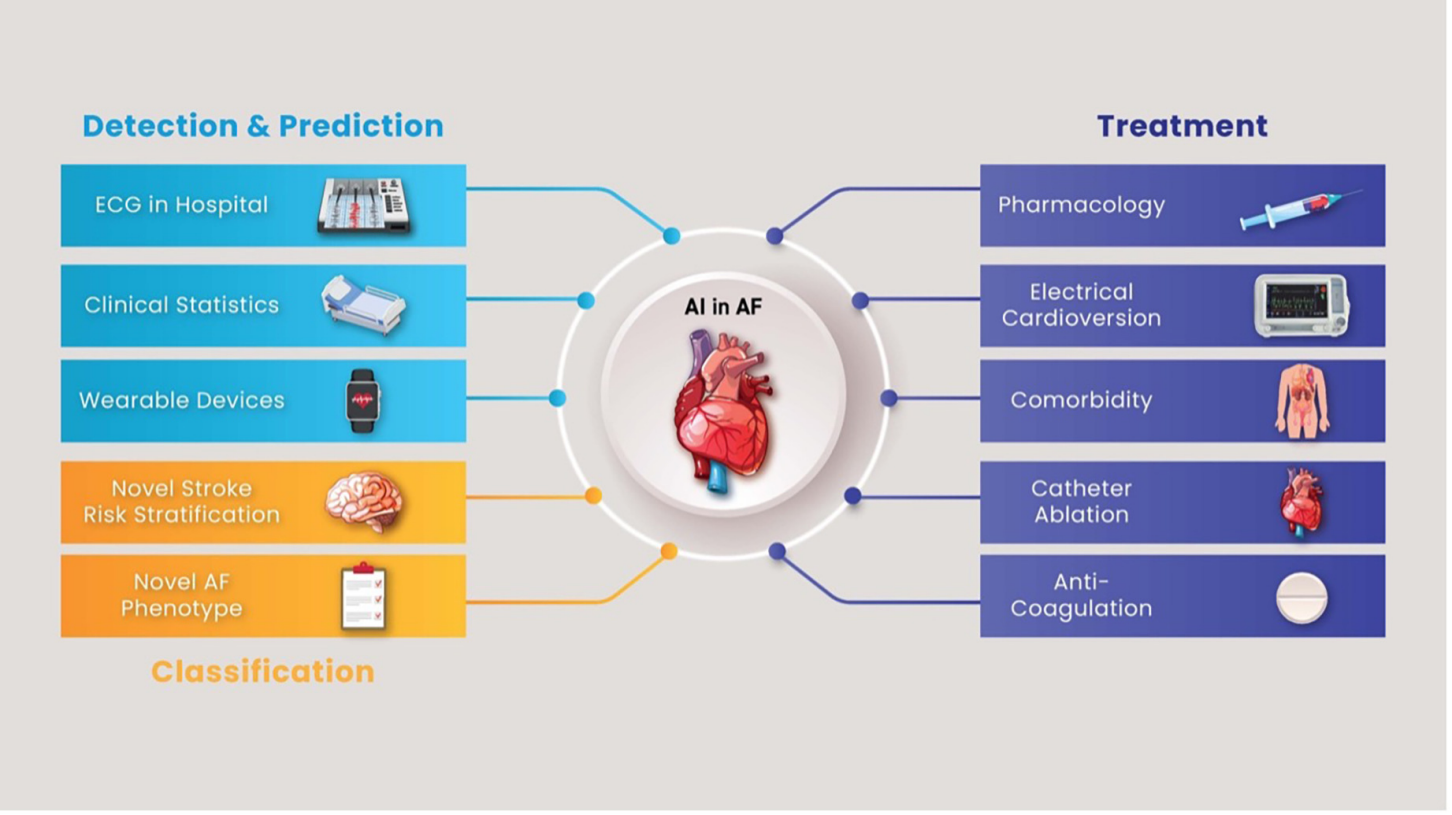
Summary of artificial use in the diagnosis and treatment of atrial fibrillation. AF,atrial fibrillation; AI, artificial intelligence; ECG, electrocardiogram.

Finally, the ultimate test for any AI application is demonstrating improved patient outcomes. Future research must include prospective, randomised trials where AI-guided care is compared to standard care in AF. These trials could examine endpoints like reduced AF burden, hospital admissions, stroke rates, or improved quality of life. For example, a trial might randomise clinics to use an AI-guided AF screening and management protocol vs. usual guideline-based management to see if the AI arm achieves better outcomes. Additionally, health economics research should evaluate the cost-effectiveness of AI interventions. Positive results will be key to convincing guidelines committees and payers to endorse AI tools in routine AF care (19). A summary of AI use in diagnosing, classifying and treating AF is demonstrated in Figure 2. The main take-home messages from this review, covering diagnostic, therapeutic, ethical, and implementation aspects of AI in AF management, are summarised in Table 4.

**Table 4:** Main take-home messages of the article.

Domain	Key take-home messages
Early detection and diagnosis	AI significantly improves early detection of AF using ECG and wearable data, but false positives and clinical follow-up remain challenges.
Risk stratification and prediction	AI-driven predictive models outperform traditional risk scores, providing precise stroke and AF progression risk assessments.
Treatment optimization	AI supports personalized decisions in anticoagulation therapy, antiarrhythmic drug selection, and catheter ablation planning, improving patient outcomes.
Remote monitoring and adherence	AI-enabled remote monitoring enhances continuous patient surveillance and adherence yet necessitates behavioural interventions for optimal clinical impact.
Ethical and Explainability concerns	Explainable AI (XAI) techniques are essential to ensure transparency, clinician trust, and ethical implementation, reducing bias and promoting equitable care.
Challenges in clinical implementation	Clinical validation, regulatory approval, integration with clinical workflows, and clinician training are crucial barriers needing attention to enable widespread AI adoption.
Future research directions	Priorities include large-scale prospective validation trials, multimodal data integration, standardizing datasets, and leveraging AI for personalized, proactive AF management.

AF, atrial fibrillation; AI, artificial intelligence; ECG, electrocardiogram; XAI, explainable artificial intelligence.

## Conclusion

In conclusion, AI holds immense potential to transform AF detection and management—from identifying the arrhythmia earlier and more accurately to personalising therapy decisions and continuously supporting patients in their daily management. The existing literature provides a robust foundation but also highlights gaps, such as inconsistent tool performance and limited clinical validation. By focusing on the identified research directions—enhancing data quality, ensuring ethical implementation, and rigorously testing AI in practice—the next wave of studies can help realise AI's promise in atrial fibrillation. In the coming years, we will see AI progress from exploratory trials into integrated clinical practice, ultimately improving outcomes and quality of life for patients with AF, provided we address the challenges and learn from ongoing research at every step. Despite promising advancements across detection, risk stratification, treatment optimisation, and remote monitoring, the clinical implementation of AI in AF remains hindered by a persistent lack of large-scale prospective RCT evidence. This limitation, common across many AI applications in medicine, highlights the need for rigorous validation to establish real-world efficacy, safety, and cost-effectiveness. Addressing this evidence gap through well-designed, multi-centre RCTs will be essential for translating AI innovations into routine clinical practice.
